# I. H. A. and Work

**Published:** 1887-08

**Authors:** P. P. Wells


### I. H. A. AND WORK.

Editors H. P.:—I have just received the July number of your journal, and find in it a hint which I wish to emphasize, viz.; that the chairmen of the bureaus of the I. H. A. organize their working forces now, and that they all begin at once on their work for the Niagara meeting.* They can never do this work so well as while under the inspiration carried away from Long Branch. It is work, and work only, by which the I. H. A. is to live and grow into the power which is prophesied of it by the experiences of its sessions of '86 and '87. It must not only be work, but it must each year bring to its gatherings better work than was found in them the year before. There is no alternative. It must do this or the Association must dwindle into incurable marasmus.

Now the year is none too long for this indispensable work; nor can it be entered on too soon, nor prosecuted with too great enthusiasm. Let those who thus early enter on this duty go over their work again and again during the year until they are models of lucidity, terseness, and logic. A year is not too much time in which to elaborate and perfect such work as the members of our bureaus should bring to the enlightenment and en-

---

* We will gladly publish the bureaux lists at once, and any further notices that the officers may desire to publish.—Eds. H. P.

largement of the knowledge of those who will gather at Niagara to receive their contributions.

Chairmen and members should feel assured now that that meeting is to be no place for the reception of commonplaces, therefore they should begin *now*, and not cease to work till the pabulum is elaborated which can alone assure life, health, and strength to the I. H. A.

P. P. WELLS.

